# Extra virgin olive oil extract rich in secoiridoids induces an anti-inflammatory profile in peripheral blood mononuclear cells from obese children

**DOI:** 10.3389/fnut.2022.1017090

**Published:** 2022-10-26

**Authors:** Stefania De Santis, Pasquale Crupi, Laura Piacente, Anna Mestice, Nicola Antonio Colabufo, Loredana Amodio, Paola Pontrelli, Loreto Gesualdo, Antonio Moschetta, Maria Lisa Clodoveo, Maria Felicia Faienza, Filomena Corbo

**Affiliations:** ^1^Department of Pharmacy-Pharmaceutical Science, University of Bari Aldo Moro, Bari, Italy; ^2^Department of Interdisciplinary Medicine, University of Bari Aldo Moro, Bari, Italy; ^3^Pediatric Unit, Department of Biomedical Sciences and Human Oncology, University of Bari Aldo Moro, Bari, Italy; ^4^Hematology Unit, Department of Emergency and Organ Transplantation, University of Bari Aldo Moro, Bari, Italy; ^5^Biofordrug, Laboratory for Clinical and Chemical Analyses, Bari, Italy; ^6^Nephrology Unit, Department of Emergency and Organ Transplantation, University of Bari Aldo Moro, Bari, Italy

**Keywords:** pediatric obesity, peripheral blood mononuclear cells (PBMCs), anti-inflammatory activity, extra virgin olive oil (EVOO), secoiridoids, tandem mass spectrometry, factor analysis

## Abstract

Obesity represents an important public health challenge of the twenty first century reaching epidemic proportions worldwide; this is especially true for the pediatric population. In this context, bioactive compounds from foods are crucial to counteract chronic inflammation as a typical feature of obesity. In particular, extra virgin olive oil (EVOO) is one of the most important functional foods exerting, among others, an anti-inflammatory activity not only due to its major (monounsaturated fatty acids) but also to its minor (phenolics) components, as reported in the last years. However, only a limited number of studies were performed on pediatric population, and even fewer are those focusing on EVOO phenolics that investigate the correlation of the chemical characterization with the biological function. Thus, starting from our *in vitro* data identifying an EVOO chemical profile characterized by a high content of secoiridoids correlating with an anti-inflammatory effect, we studied the ability of an EVOO extract with the same chemical profile to retain this function *ex vivo*. Specifically, peripheral blood mononuclear cells (PBMCs) collected from obese children were treated with EVOO and olive oil extracts, characterized by a low polyphenol content, to study the ability of secoiridoids to dampen the inflammatory response. A reduction of pro-inflammatory CD14^+^CD16^+^ monocytes was detected by cytofluorimetric analysis when PBMCs were treated with EVOO as compared to olive oil extracts. According to this, a down modulation of CCL2 and CCL4 chemokines involved in the recruitment of inflammatory cells, was reported in the supernatants of EVOO relative to olive oil extracts treated PBMCs. Moreover, a high-throughput gene expression analysis revealed that PBMCs molecular profile from obese children is greatly modulated after the treatment with EVOO extract in terms of metabolic and inflammatory pathways. Importantly, some of the significantly modulated genes were involved in the pathways promoting the development of severe obesity. Overall, our *ex vivo* data demonstrated the ability of EVOO to reduce the inflammatory *milieu* of PBMCs from obese children both at protein and molecular levels. Of note, a good correlation between the EVOO chemical profile and the biological modulations in terms of anti-inflammatory activity was reported.

## Introduction

Obesity is a complex multifactorial disease reaching epidemic proportions worldwide thus becoming one of the most important public health challenges of the twenty first century (1). In fact, it has been identified as a major determinant of disability and death due to the related increase in the risk of non-communicable diseases (1). This is alarming especially considering the even faster rate for school-age and adolescents with obesity reported in the last decades (1). A multi-disciplinary approach based on the promotion of a healthy lifestyle characterized by an increased physical activity and the adoption of a dietary regimen enriched in fruit, vegetables, legumes, cereals, and nuts, with a low content of free sugars, eggs, salt, and saturated fats has been proposed (i.e., Mediterranean Diet, MD) to tackle the huge increase in childhood obesity incidence and its health implications (2, 3). Specifically, the recent literature supports a crucial role for bioactive compounds from foods in the modulation of chronic inflammation as a typical feature of chronic diseases, including obesity (4–6). Among the well-studied functional foods characterized by health-promoting properties, there is the extra virgin olive oil (EVOO) to which many of the MD beneficial effects could be ascribed thanks to a synergic effect of monounsaturated fatty acid and phenolic compounds, identified as major and minor components, respectively (5, 7). The positive effects on human health for EVOO phenolics were greatly re-evaluated in the last years (7). However, even if the antioxidant, anti-inflammatory, and anti-cancer properties of EVOO phenolic compounds were demonstrated in many papers on obesity (8, 9), only a limited number of studies were conducted on a pediatric population (10–12). Importantly, to our knowledge, studies using EVOO phenolic compounds that demonstrate a correlation between their chemical characterization and their biological activity are still limited to date (13, 14). This consideration could also be extended to studies on a pediatric population and, specifically, on childhood obesity. Therefore, starting from our previous identification of a specific chemical profile for an EVOO extract characterized by a group of polyphenols, mainly secoiridoids, acting synergistically to promote an anti-inflammatory activity in an *in vitro* study, in this paper we aim to verify if an EVOO extract with the same chemical profile is able to retain this beneficial property *ex vivo* in the context of childhood obesity (15). Thus, we studied the modulation of the inflammatory response at protein and molecular levels in peripheral blood mononuclear cells (PBMCs) isolated from obese children after the treatment with an EVOO extract as compared to a rectified olive oil extract, characterized by a low content of polyphenols, to demonstrate the anti-inflammatory activity of EVOO phenolics in childhood obesity. Moreover, we also demonstrated a correlation between the phenolic profile (determined by HPLC-MS/MS analyses) of EVOO and rectified olive oil extracts, and the biological modulations of the inflammatory pathways both at protein and molecular levels, thus bridging the gap between the EVOO chemical profile and its biological effect in terms of anti-inflammatory activity.

## Materials and methods

### Olive oil samples

Secoiridoids-rich EVOO and rectified oil samples (hereafter indicated as EVOO and olive oil, respectively) were produced in the Apulian region (Italy) during the 2021/2022 campaign using olives from Coratina cultivar.

### Extraction phenolic compounds from olive oil samples

Phenolic compounds were extracted from the selected olive oil samples (2 g), carefully weight into end capped amber vials in presence of 5 mL methanol/water solution 80:20 (v/v), by using an ultrasonic water bath (Elmasonic P 30H, Elma Schmidbauer GmbH, Singen, Germany) for 15 min at room temperature. After the ultrasound treatment, the extracts were centrifuged at 4,400 × g for 25 min in an Eppendorf centrifuge 5810R (Hamburg—Germany) and filtered through a 0.20 μm syringe regenerated cellulose filters (VWR International Srl, Milano, Italy) before the HPLC-MS/MS analyses.

### Study participants

A total number of 13 obese children (6 male, 7 women) with a mean age of 9.85 ± 2.6 years were enrolled at the Endocrinology Unit of Pediatric Hospital Giovanni XXIII, Aldo Moro University of Bari. Inclusion criteria were body mass index (BMI) ≥ 95th percentile for age and sex. Exclusion criteria were type 2 diabetes mellitus, secondary or syndromic forms of obesity, hypothyroidism, Cushing disease, viral hepatitis, metabolic or genetic liver diseases, and ongoing therapies for chronic systemic diseases. All subjects were in a good general health and were not taking drugs in the last 3 months. The study involving human participants was conducted in compliance with the Helsinki Declaration and reviewed and approved by the Independent Ethical Committee of Azienda Ospedaliera-Universitaria “Consorziale Policlinico” of Bari. Written informed consent to participate in this study was provided by the participants’ legal guardian/next of kin.

### Isolation and culture of peripheral blood mononuclear cells from obese children

PBMCs were isolated by centrifugation of peripheral blood samples over Histopaque 1,077 density gradient (Sigma Chemical, St. Louis, MO), and cultured in RPMI-1640 (Thermo Fisher Scientific, Waltham, MA, USA) supplemented with 10% heat-inactivated fetal bovine serum (FBS, Thermo Fisher Scientific, Waltham, MA, USA), 100 U/mL penicillin and 100 mg/mL streptomycin (Thermo Fisher Scientific, Waltham, MA, USA) at 37°C in a humidified 5% CO_2_ atmosphere. Isolated PBMCs were separately cultured in presence of control (methanol, indicated as Ctrl), EVOO and olive oil extracts (6.25 μg/mL each) to perform both gene expression and chemokines quantification at 6 h, and cytofluorimetric analysis at 24 h. The supernatants of PBMCs cultured with Ctrl, EVOO, and olive oil extracts were collected following microcentrifugation at 1,700 × g for 10 min and immediately stored at –80°C until use for chemokines quantification and HPLC-MS/MS analyses.

### Gene expression analysis by real-time polymerase chain reaction

Total RNA was isolated from PBMCs of 9 obese children (5 male, 4 women) after 6 h of treatment with Ctrl, EVOO and olive oil extracts, using TRIzol^®^ (Thermo Fisher Scientific, Waltham, MA, USA) according to the manufacturer’s instructions. Total RNA (1 μg) was reverse transcribed using an iScript cDNA Synthesis kit (Biorad, Hercules, CA, USA) with random primers for cDNA synthesis. High throughput molecular analysis was performed using PrimePCR^®^ plates (cat #: 10035852, Biorad, Hercules, CA, USA) on a CFX96 System instrument (Biorad Laboratories, Hercules, CA, USA).

### Chemokines quantification by ProQuantum^®^ immunoassay kits

Six hours after the treatment with EVOO and olive oil extracts, supernatants from PBMCs cultures of 10 patients (5 male, 5 women) were collected and used to quantify the expression of C-C chemokine ligand type 2 (CCL2, also indicated as monocyte chemoattractant protein-1, MCP-1) and CCL4 (also indicated as macrophage inflammatory protein-1β, MIP-1β) chemokines by a high-sensitivity immunoassays kit using Real-Time PCR as a readout, according to the manufacturer’s instructions (cat. #: A35598 and A35597, respectively; Thermo Fisher Scientific, Waltham, MA, USA). ProQuantum^®^ software (apps.thermofisher.com/apps/proquantum) was used to generate standard curves and determine sample concentrations.

### Cytofluorimetric analysis

PBMCs isolated from 13 obese children (6 male, 7 women) were treated with Ctrl, EVOO and olive oil extracts for 24 h. After the treatment, PBMCs were detached from the plate with cold D-PBS 1X (Gibco, New York, NY, USA) + 0.5 mM EDTA (Thermo Fisher Scientific, Waltham, MA, USA), washed with D-PBS 1X + 2 mM EDTA (Thermo Fisher Scientific, Waltham, MA, USA) + 0.5%BSA (Sigma-Aldrich, St Louis, MO, USA), and stained with CD14 FITC/CD64 PE (Becton, Dickinson and Company, OR, USA, REF:333179), CD16 APC and CD45 VioGreen^®^ (Miltenyi Biotec, Bergisch Gladbach, Germany, REF:130-113-951 and REF:130-110-776, respectively) according to the manufacturer’s instructions. To test cell viability, 7-AAD PerCP-Cy5.5 (7-Aminoactinomycin D, Becton, Dickinson and Company, OR, USA) was used. Flow cytometer acquisition was performed using the FACSCanto II flow cytometer (Becton, Dickinson and Company, OR, USA), and data were analyzed using Diva software, version 8.0.1 (Becton, Dickinson and Company, OR, USA). Gating strategy: after the selection of single and live cells from PBMCs, cells were checked for positivity to CD64 and CD45 (each marker vs. side scatter). CD64^+^ CD45^+^ cells were analyzed for the expression of CD16 and CD14 by a dot plot (Supplementary Figure 1).

### HPLC-MS/MS analyses

HPLC-MS/MS analyses were performed on cultured PBMCs treated with EVOO and olive oil after 6 and 24 h and compared to the supernatants collected immediately after the treatment (time 0). Before to start with these analyses, the supernatants from PBMCs were simply filtered through a 0.20 μm syringe regenerated cellulose filters (VWR International Srl, Milano, Italy). Separation and identification of the compounds were performed on a Shimadzu LC-MS 8040 T ri p Q u a d M S equipped with System Controller CBM-20A, Trip Quad Mass Spectrometer detector, and controlled by LabSolutions WS Software (Neonatal Solution version 2.0 part-number 225-24442-91). The chromatography was accomplished through a reversed stationary phase Zorbax Extend-C18 (50 × 2.1 mm i.d., particle size 1.8 μm, Agilent Technologies, Palo Alto, CA, USA) protected by a C18 Guard Cartridge (4.0 × 2.0 mm i.d., Phenomenex) and a binary mobile phase composed of solvent A (water containing 0.1% (v/v) formic acid) and solvent B (acetonitrile, Chromasolv, VWR International Srl, Milano, Italy). The following gradient was adopted: 0 min, 10% B; 0.33 min, 10% B; 5 min, 30% B; 7.33 min, 50% B; 9.33 min, 100% B; then the column was re-equilibrated with ∼20 column volume. The column temperature was controlled at 25°C, the flow was maintained at 0.44 mL/min, and the injection volume was 1 μL. Ionization of the molecules was acquired in negative ESI mode with capillary voltage at 4,000 V, using nitrogen as drying (*T* = 250°C; flow rate = 15 L/min) and nebulizing gas (3 L/min). The mass acquisition in MS and MS/MS spectra ranged between *m/z* 50 and 1,200. Typically, two runs were performed during the HPLC-ESI-MS analysis of each sample. First, an MS full-scan acquisition was performed to obtain preliminary information on the predominant *m/z* ratios observed during the elution and then MS/MS spectra were acquired. Tentative compound identification was achieved, similarly to that reported in our previous study (15), by comparing mass spectra (MS and MS/MS) with those from pure standards, when available, and/or interpreted with the help of structural models already hypothesized in the literature. Then, the main revealed compounds were quantified by multiple reaction monitoring (MRM) as 3-hydroxy-tyrosol (*R*^2^ = 0.9976; LOD = 0.75 μg/g; LOQ = 2.5 μg/g), oleuropein (*R*^2^ = 0.9982; LOD = 0.98 μg/g; LOQ = 3.25 μg/g), luteolin (*R*^2^ = 0.9997; LOD = 0.06 μg/g; LOQ = 0.20 μg/g), and apigenin (*R*^2^ = 0.9942; LOD = 0.075 μg/g; LOQ = 0.25 μg/g) equivalents, depending on their structural similarity (Supplementary Table 1). The detection limit (LOD) and quantification limit (LOQ) were calculated from the calibration curves (3 and 10 folds, respectively, the ratio between intercept error and slope).

### Statistical analysis

Statistical analysis of biological data was performed using GraphPad Prism statistical software release 9.3.1 for Windows XP. Data were expressed as means ± SEM obtained from all patients for each experimental group. For cytofluorimetric analysis, the statistical significance of CD16 median fluorescence was calculated by Kruskal-Wallis test and Dunn’s multiple comparison test while the statistical significance of cell viability was evaluated using one-way ANOVA test. For chemokines analysis, two-tailed Mann-Whitney test was used to check statistical significance. The analysis of PBMCs molecular profile was performed using the Bio-Rad CFX Maestro 2.3 software release 5.3.022.1030; for the Volcano Plot, the statistical significance was calculated using an unpaired *t*-test. A Fold Change Threshold (calculated as Fold Regulation) ≥ 2.40 was set. Differentially expressed genes were functionally analyzed using the IPA software.^1^ All the biological results were considered statistically significant at *p* < 0.05. Data from HPLC-MS/MS analyses of polyphenols in the cellular supernatants at 0, 6, and 24 h of PBMCs treated with olive oil or EVOO were analyzed using the STATISTICA 12.0 software package (StatSoft Inc., Tulxa, OK, USA). Specifically, a two-way factorial analysis of variance (ANOVA), followed by Tukey’s HSD *post-hoc* test was carried out in order to evaluate the significant different means (*p* < 0.05). Subsequently, a factor analysis (FA) with orthogonal rotation of axes (varimax rotation) was performed on logarithmic and Pareto-scaling transformed data of polyphenols (calculated as difference between 0 and 6 h in the cellular supernatants), mRNAs (determined at 6 h), and proteins (determined at 6 and 24 h).

## Results

### Characterization of extra virgin olive oil and olive oil phenolic extracts

Twenty polyphenols, including one simple phenol, sixteen secoiridoids, and three flavonoids, were identified in the oil extracts by comparing MS characteristics ([M-H]^–^ deprotonated ions and MS/MS fragments) to those reported in De Santis et al. (15). The amounts of these compounds were subsequently determined by MRM experiments and expressed as μg/g of oil (Supplementary Table 1). As expected, they were more concentrated in EVOO than olive oil. In particular, 3-hydroxytyrosol, luteolin, apigenin, and methoxyluteolin were from 70 to 150 folds more concentrated and ligstroside aglycone forms reached values up to 180 folds higher in EVOO than olive oil. Moreover, the two oleuropein isomers and oleochantalic acid were only present in EVOO samples.

### Modulation of CD14^+^CD16^+^ pro-inflammatory cells in peripheral blood mononuclear cells from obese children after extra virgin olive oil treatment

**FIGURE 1:**
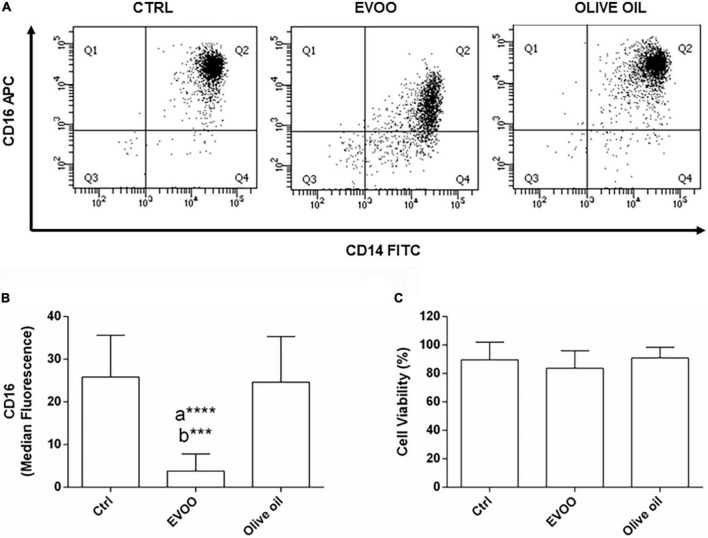
EVOO extract significantly reduced the expression of CD16 molecule among the pro-inflammatory CD14^+^CD16^+^ monocyte population in PBMCs from obese children. **(A)** Representative dot plot of CD14 and CD16 expression on monocytes (CD64^+^CD45^+^) from PBMCs, each treated for 24 h with Ctrl, EVOO, and olive oil extracts (6.25 μg/mL each). **(B)** Histogram represents CD16 cell surface expression calculated on CD14^+^CD16^+^ cell population. Data are expressed as a mean of median fluorescence ± SEM (*n* = 13). **(C)** Histogram indicates the percentage of cell viability ± SEM (*n* = 13) in the experimental groups. a: EVOO vs. Ctrl; b: EVOO vs. olive oil ****p < 0.0001, ***p < 0.001.

With the intent to verify if the chemical EVOO profile selected from our *in vitro* study for its anti-inflammatory activity retains this biological function *ex vivo*, PBMCs from obese children were cultured with an EVOO extract characterized by the same chemical profile. To study the ability of EVOO extract to modulate the expression of circulating mononuclear cells involved in chronic diseases such as obesity, the expression of CD14^+^CD16^+^ cell population was evaluated by cytofluorimetric analysis among the monocyte population (CD64^+^CD45^+^ cells; gating strategy is reported in Supplementary Figure 1). Specifically, the *ex vivo* treatment of PBMCs for 24 h with EVOO extract was able to significantly reduce the median fluorescence of CD16 as surface protein, in the CD14^+^CD16^+^ cell population as compared to Ctrl and olive oil extract (Figures 1A,B) without affecting cell viability (Figure 1C). Of note, no modulation was detected comparing olive oil extract with Ctrl sample (Figures 1A,B).

### Molecular profile of peripheral blood mononuclear cells from obese children induced by extra virgin olive oil treatment

**FIGURE 2:**
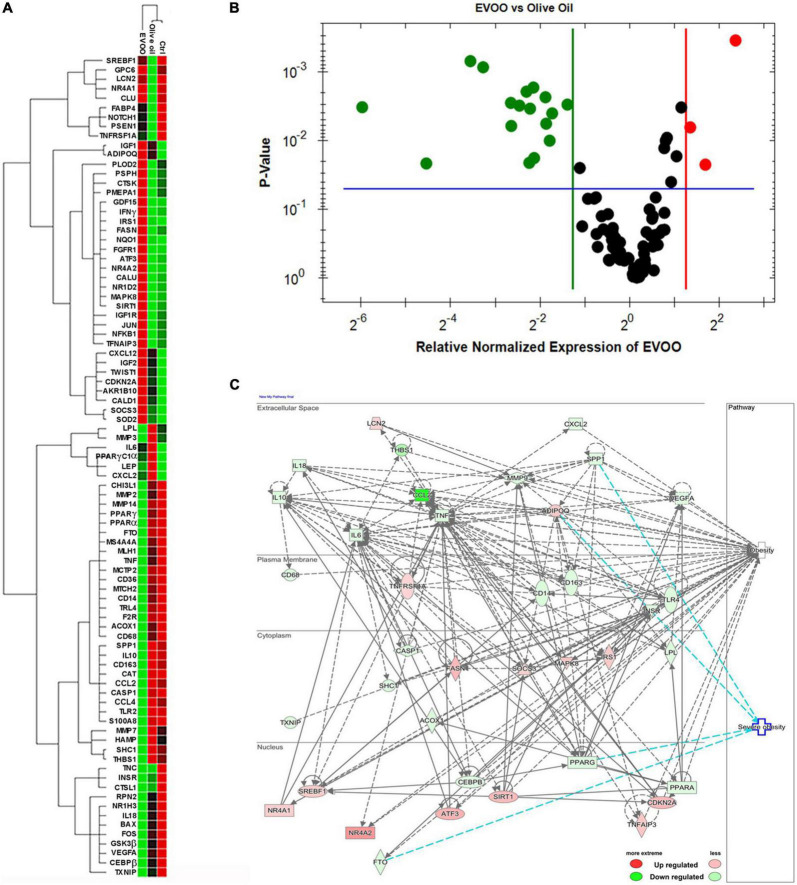
The molecular profile of PBMCs from obese children is greatly modulated after the treatment with EVOO extract in terms of metabolic and inflammatory pathways. **(A)** Hierarchical clustering using the differentially expressed genes from PBMCs separately treated with EVOO, olive oil, and Ctrl for 6 h (6.25μg/mL; *n* = 9). **(B)** Volcano plot representation of differential expression analysis of genes in EVOO vs. olive oil treated PBMCs (*p* < 0.05 and fold change threshold ≥ 2.4). **(C)** Networks analysis of obesity and severe obesity pathways generated by IPA for the differentially expressed genes between PBMCs treated with EVOO as compared to olive oil.

RNA from PBMCs treated *ex vivo* for 6 h with EVOO and olive oil extracts relative to Ctrl sample was analyzed by qPCR to measure the expression of 89 selected genes specific for the obesity pathway. Hierarchical clustering of data showed a distinct segregation among EVOO extract treated PBMCs relative to olive oil extract and control treated PBMCs that, in line with the cytofluorimetric data, segregated together (Figure 2A).

**FIGURE 3:**
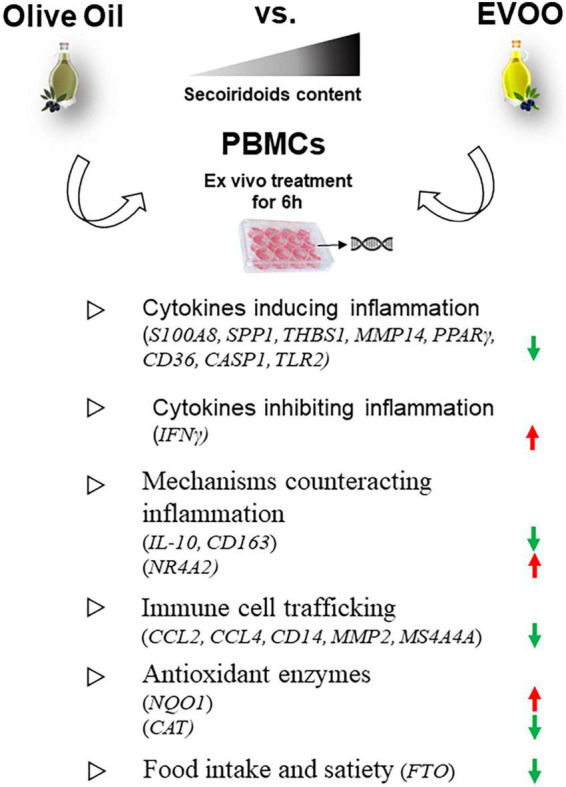
Summary of the molecular modulations along with the relative pathways induced by a 6-h treatment of PBMCs with EVOO compared to olive oil extracts. Arrow direction indicates the up- or- down modulation of genes in PBMCs treated with EVOO using those treated with olive oil as reference. Green arrows: down modulated genes; red arrows: up modulated genes.

Moreover, the Volcano Plot identified 3 significantly up modulated genes (*NQO1, IFN*γ, *NR4A2*, red dots in Figure 2B) and 17 significantly down modulated genes (*CCL2, THBS1, S100A8, CD14, MMP2, SPP1, MS4A4A, PPAR*γ, *CD163, CASP1, TLR2, CCL4, FTO, MMP14, CD36, CAT, IL-10*, green dots in Figure 2B) when the PBMCs were treated with EVOO extract as compared to olive oil extract. A summary of these molecular modulations along with the related pathways, is reported in Figure 3.

The analysis by IPA of the main biological and pathological functions in which the differentially expressed genes of PBMCs treated with EVOO vs. olive oil were included, indicated metabolic diseases (*p* range = 1.85E-19/3.76E-40, 63 genes), inflammatory response (*p* range = 3.84E-19/1.20E-39, 74 genes), and immune cell trafficking (*p* range = 3.84E-19/3.20E-44, 63 genes) as biological pathways (data not shown).

IPA analysis also revealed that most of the modulated genes after EVOO treatment, are molecules that can influence the obesity signaling pathway (Figure 2C). Moreover, IPA analysis also revealed a *niche* of genes that could be specifically linked to severe obesity. Specifically, the secreted *SPP1* and the nuclear *PPAR*γ and *FTO* were included among the down modulated genes associated to severe obesity pathway after PBMCs treatment with EVOO, while ADIPOQ was up regulated (Figure 2C).

### Early modulation of chemokines expression after the treatment of peripheral blood mononuclear cells from obese children with extra virgin olive oil

**FIGURE 4:**
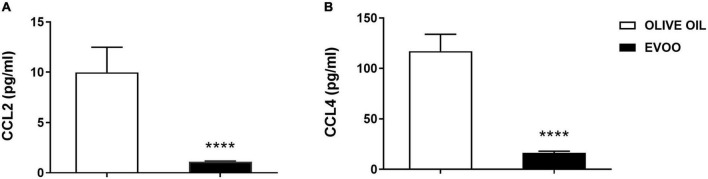
EVOO extract significantly reduced the expression of important chemokines at protein level as early as 6 h after PBMCs treatment relative to olive oil. Histograms represent CCL2 **(A)** and CCL4 **(B)** protein expression tested by a high-sensitivity immunoassay using Real-Time PCR as a readout, in the supernatant of PBMCs individually treated with EVOO or olive oil for 6 h (6.25μg/mL). Data are expressed as a mean of triplicate concentration values ± SEM of two independent experiments (*n* = 10). ****p < 0.0001.

Since after 6 h of EVOO treatment we observed a significant down modulation of some chemokine genes, i.e., *CCL2* and *CCL4*, we also tested if this treatment could impact on the protein level of these pro-inflammatory chemokines, at the same time point. To this aim, we tested the expression of these chemokines as secreted proteins in PBMCs supernatants 6 h after the treatment with EVOO and olive oil extracts. Due to the paucity of cell material recovered from pediatric samples, we performed a high-sensitivity immunoassay using Real-Time PCR as a readout because it requires a small quantity of supernatants for the analysis if compared to conventional methods analyzing proteins. We demonstrated that EVOO extract was able to reduce both CCL2 (Figure 4A) and CCL4 (Figure 4B) also at protein level as early as 6 h after PBMCs treatment when compared to olive oil.

### Characterization of cellular supernatants treated with olive oil extracts by HPLC-MS/MS analyses

With the intent to correlate the biological effect with oil extracts phenolic composition, we characterized the cellular supernatants of PBMCs treated with EVOO and olive oil by HPLC-MS/MS analyses. To simulate real samples, the control was spiked with a methanolic solution containing phenolics standards commercially available (i.e., 3-hydroxytyrosol, oleuropein, luteolin, and apigenin) at a similar concentration to that found in olive oil extracts (Supplementary Figure 2). Because the good recoveries (around 90% for all the compounds) obtained after HPLC-MS/MS analyses lead to excluding significant matrix interference or ion suppression phenomena, the cell supernatants were only filtered and directly injected without any extraction procedure. Spiked control samples were also used to optimize chromatographic and mass spectrometry conditions as well as to construct calibration curves. The polyphenols identified in the cell medium treated with olive oil extracts were quantified in the supernatants, collected immediately after the treatment (time 0) and at 6 and 24 h from the treatment, to monitor the possible cellular uptake. Although they have different chemical structures, both simple phenols and secoiridoids were not revealed in the supernatants at 6 h (Supplementary Table 2). No accumulation of 3-hydroxytyrosol and the absence of tyrosol (deriving from the decomposition of oleuropein and ligstroside isomers, respectively) were observed both at 6 and 24 h. On the other hand, the behavior of the flavonoids was in sufficient agreement with the literature, indeed apigenin and methoxyluteolin (presenting a lower degree of hydroxylation) showed more limited diffusion into the cell than luteolin (16). However, no significant differences were reported in flavonoid content between 6 and 24 h (Supplementary Table 2).

### Correlation between extra virgin olive oil phenolic compounds and anti-inflammatory response in peripheral blood mononuclear cells from obese children

**FIGURE 5:**
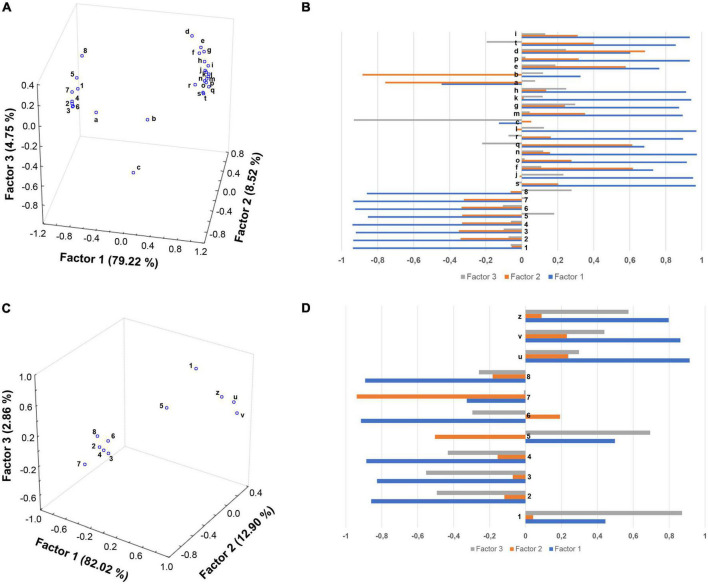
Factor analysis indicates a good correlation between the EVOO chemical profile and the biological modulations. Score plots **(A,C)** and factor loadings **(B,D)** relative to oil polyphenols and gene expression data at 6 h **(A,B)**, and protein data at 6 and 24 h **(C,D)** were reported. Olive oil polyphenols: (1) 3-hydroxytyrosol; (2) oleuropein isomers; (3) oleuropein aglycone forms; (4) oleochantalic acid; (5) luteolin; (6) ligstroside aglycone forms; (7) apigenin; (8) methoxyluteolin. mRNAs: (a) *NQO1*; (b) *NR4A2*; (c) *INF*γ; (d) *SSP1*; (e) *PPAR*γ; (f) *CCL2*; (g) *MMP14*; (h) *MS4A4A*; (i) *TLR2*; (j) *CAT*; (k) *MMP2*; (l) *FTO*; (m) *IL-10*; (n) *CD14*; (o) *CCL4*; (p) *S100A8*; (q) *CD163*; (r) *CD36*; (s) *CASP1*; (t) *THBS1*. Proteins 6 h: (u) CCL2 and (v) CCL4. Proteins 24 h: (z) C14^+^C16^+^ cell population.

To tentatively discover a possible correlation between the phenolic compounds and anti-inflammatory response in PBMCs from obese children, FA analyses were performed on polyphenols in the supernatants of cells treated with EVOO and olive oil extracts (computed as difference between 0 and 6 h) and mRNAs (determined at 6 h, Figures 5A,B) or proteins (determined both at 6 and 24 h; Figures 5C,D). Among the polyphenols analyzed, oleuropein aglycones and ligstroside aglycones values were summed together. Data for FA analyses were edited using the logarithmic transformation and Pareto-scaling methods to reduce the relative importance of large values but keep the data structure partially intact (17). On the basis of Kaiser rules and scree test, three factors were extracted to explain the maximum variance in the dataset (92.49 and 97.78%) of both FAs without losing too much information. Furthermore, the orthogonal axes rotation (varimax rotation) was applied for obtaining a clearer factor loadings pattern and enhancing the interpretability of the dataset structures (Figure 5). Regarding the first FA score plot, Factor 1 retained the main explained variance (79.22%), with all the polyphenols and the great part of gene transcripts having a high negative (close to –0.9) and positive (ranging between 0.73 and 0.97) loadings (Figures 5A,B). This could suggest that both 3-hydroxytyrosol, oleuropeins, ligstrosides, and flavonoids (more concentrated in EVOO extract treated cells) played a synergistic role in the down regulation of genes involved in the inflammatory response, such as those associated with severe obesity pathway (*SPP1, PPAR*γ, and *FTO*). Conversely, the genes *NQO1* (Figures 5A,Ba), *NR4A2* (Figures 5A,Bb), and *INF*γ (Figures 5A,Bc), mainly projected on the orthogonal Factor 2 (i.e., *NQO1* and *NR4A2* with factor loadings of –0.7583 and –0.8839, respectively) and Factor 3 (*INF*γ having a loading of –0.9325), seem to be unaffected by EVOO polyphenols. About the second FA score plot, inflammatory protein expression was inversely related to few polyphenols (i.e., secoiridoids and methoxyluteolin) as proved by their opposite factor loadings (> | 0.8|) on Factor 1, accounting for 82.02% of the explained variance (Figures 5C,D). However, surprisingly with respect to the previous analysis, apigenin (mainly projected on Factor 2), and 3-hydroxytyrosol and luteolin (mainly projected on Factor 3) did not seem to show any significant influence on the reduction of CCL2, CCL4, and CD14^+^CD16^+^ cell population expression.

## Discussion

Numerous recent papers highlight the link between bioactive components from EVOO, mainly polyphenols, and chronic pathologies with a low-grade inflammatory state, such as obesity (8, 9). However, focusing our attention on pediatric population, the number of papers remarking the anti-inflammatory effects of EVOO became very narrow, especially when the correlation between the chemical characterization and the biological effect was taken into account (13). Thus, we decided to study this specific correlation in PBMCs isolated from obese children exposed to EVOO treatment. Starting from our *in vitro* data identifying a defined EVOO chemical profile correlating with an anti-inflammatory effect (15), we studied the ability of an EVOO extract with the same chemical profile (rich in secoiridoids) to modulate the inflammatory *milieu ex vivo*. Specifically, PBMCs collected from obese children were treated with secoiridoids-rich EVOO extract and olive oil extract, characterized by a low polyphenol content, to study the ability of polyphenols to dampen the inflammatory response. Based on our results, the *ex vivo* treatment with EVOO was able to reduce the inflammatory *milieu* characterizing PBMCs isolated from obese children. Specifically, we identified a reduction of CD16 protein expression among the monocyte population 24 h after EVOO treatment as compared to olive oil. Importantly, the latter shows the same expression of Ctrl sample thus emphasizing the role of polyphenols for this biological function. This result is in line with several studies reporting a significant increase in CD14^+^CD16^+^ cell population in chronic inflammatory diseases, such as obesity (12, 18–20). In fact, this cell population shows a macrophage-like phenotype with enhanced antigen-presenting ability and is able to secrete pro-inflammatory cytokines [like TNF, (21)], and chemokines, such as CCL3 (also known as MIP-1α) and CCL4 (22, 23). Moreover, CD14^+^CD16^+^ cells display different chemokine-receptor expression profiles, potentially reflecting distinct recruitment properties. Opposite to CD14^+^CD16^–^, CD14^+^CD16^+^ monocytes are CCR2 negative and express high levels of CCR5 and CX3 chemokine receptor-1 (CXCR1, the fractalkine receptor) (24) that mediate the chemotaxis of immune cells. These data are in line with our results demonstrating a reduced quantity of CCL2 both at molecular and protein levels. Furthermore, *CCL2* is the most down regulated gene in PBMCs from obese children treated with EVOO (FC = –62.84, *p* = 0.003) and its protein expression resulted decreased in PBMCs supernatants as early as 6 h after EVOO treatment when compared to olive oil. In accordance with the chemokines profile for CD14^+^CD16^+^ cell population reported in literature, we demonstrated a down modulation of *CCL4* (FC = –4.39, *p* = 0.01) and its secreted protein, with an about 10-fold increase of CCL4 protein expression relative to CCL2 6 h after EVOO treatment (CCL2: 9.98 pg/mL; CCL4: 117.01 pg/mL).

Moreover, the polyphenols’ role in promoting the anti-inflammatory effect also came up looking at the molecular data. In fact, the hierarchical clustering on Real-Time PCR gene expression data studying the PBMCs molecular profile showed a distinct segregation between EVOO and olive oil when compared to untreated sample. Thus, we decided to focus on the comparison between EVOO and olive oil extracts. Specifically, the Volcano Plot identified the down modulation of some genes involved in the secretion of pro-inflammatory cytokines in obesity context, such as *S100A8* (25), *SPP1* (26), *THBS1* (27), and *MMP14* (28, 29). Moreover, a reduction of *CD36* mediating Nfκb activation and, consequently, the transcription of pro-inflammatory cytokines (30), is reported in response to EVOO treatment. This mechanism could be linked to *PPAR*γ down modulation reported after EVOO treatment because CD36 is a target gene of PPARγ; moreover, the latter is important in obesity pathogenesis also due to its involvement in lipid metabolism (31). The down modulation of chemokines [*CCL2* (32) and *CCL4* (33)] and other genes promoting the recruitment and differentiation of macrophages [*SPP1* (34), *THBS1* (27), indirect mechanism of *MMP14* mediated by *MMP2* (28), and *MS4A4A* (35, 36)], and T lymphocytes [*S100A8* (25) and *THBS1* (37)] was also reported after EVOO treatment relative to olive oil. Moreover, EVOO induced down modulation of the anti-inflammatory cytokine IL-10 and the CD163 molecule, expressed on the surface of anti-inflammatory macrophages M2 in association with CD14. These results could be intended as counteractive mechanisms in chronic inflammatory conditions; in fact, both these molecules were reported to be up modulated in obese patients (38, 39).

Furthermore, it could also be ascribed to an early evaluation of EVOO treatment in obese children; this aspect represents a potential limitation of our study. Thus, additional studies are required to investigate the role of a prolonged EVOO treatment in this chronic disease.

Among the genes included in the inflammatory response we also observed the up regulation of *IFN*γ and *NR4A2* in patients treated with EVOO. Even if some papers reported a pro-inflammatory function for both these genes (40, 41), some anti-inflammatory roles are also depicted that could explain our results. Specifically, *IFN*γ indirectly inhibits the pro-inflammatory cytokine IL-18 whose mature form was processed by CASP1 starting from pro-IL-18 (42). In line with this, we found the *CASP1* is reduced after EVOO treatment as compared to olive oil. As concern *NR4A2* up modulation, it could induce Foxp3 expression as marker of T regulatory cell population (43).

Furthermore, the reduction of *TLR2* demonstrated the ability of EVOO treatment to reduce the inflammatory response activation induced by a wide range of pathogen-associated molecular patterns (PAMPs) derived from bacteria, fungi, parasites, and viruses (44, 45).

Among the analyzed genes, those linked to the oxidative pathway are the two antioxidant enzymes *NQO1* and *CAT*, showing an opposite modulation (up- and down- modulated, respectively) after EVOO treatment. Our findings on NQO1 are in line with the literature; in fact, acting as a superoxide scavenger, NQO1 preserves cells from the oxidative stress and is implicated in protection against obesity and its related comorbidities (46). On the contrary, the reduction reported for *CAT* after EVOO treatment needs further investigation in the context of obesity for a better integration of our data with the relative literature (47). To this aim, could be useful to investigate if the gene expression correlates to the enzyme activity; this is not the case after EVOO supplementation in PBMCs from healthy subjects (48). A possible explanation could be that the increase of antioxidant levels induced by NQO1 up modulation, makes CAT expression less necessary.

Interestingly, most of the modulated genes were shared among some of the important biological pathways identified by IPA analysis; metabolic diseases, inflammatory response, and immune cell trafficking, featuring the role of inflammation in promoting the pathogenesis of obesity [as for S100A8 (49) and CCL2 (50)] and its related comorbidities [as for CD163/CAT and insulin resistance (38, 51), CCL2/IL-10/IFNγ and metabolic syndrome (52–54), MMP2 and hypertension (55), and IL-10 and hypertriglyceridemia (56)], in pediatric population. Even if some of the modulated genes were associated to pediatric obesity, to our knowledge none of them was reported in literature as result of EVOO extract treatment in PBMCs from obese children so far.

In line with the biological pathways reported above, IPA analysis confirmed that the modulated genes were linked to obesity pathway and, in addition, some of these genes (*NR4A2, PPAR*γ, and *FTO*) were correlated with the pathway promoting the development of severe obesity. In fact, FTO is an important gene in obesity pathogenesis due to its ability to modulate food intake and satiety perception; however, these modulations are often linked to a genetic polymorphism (57) rather than to gene expression. This consideration could be extended to other genes such as PPARγ (58). Since genetic polymorphisms can influence gene expression, another limitation related to this point is that we haven’t analyzed the genetic background of the population enrolled in the study.

Importantly, in this paper, we investigated the correlation of EVOO chemical characterization with its biological function in terms of anti-inflammatory activity trying to fill the gap present in literature on this topic. It is worth pointing out that both simple phenols and secoiridoids were not detected in PBMCs supernatants as early as 6 h (Supplementary Table 2). Evidently, the significant reduction of secoiridoids could be also due to their spontaneous degradation in free cells cultivar medium (16). However, no accumulation of 3-hydroxytyrosol and the absence of tyrosol (deriving from the decomposition of oleuropein and ligstroside isomers, respectively) were observed at 6 and 24 h. Therefore, the decrease in the amount of these compounds in the supernatants from 6 to 24 h might indicate the uptake of intact compounds by the cells.

In line with our previous work (15), data from FA demonstrated that the presence of polyphenols and, in particular, secoiridoids in EVOO extract is fundamental to express an anti-inflammatory activity. In fact, FA analyses showed that a high concentration of polyphenols, as revealed in EVOO with respect to olive oil extracts, had a relevant effect in down regulating the genes involved in the inflammatory response. The exceptions to this trend were represented by *NQO1, NR4A2*, and *IFN*γ. The reason for the different trend reported in FA analysis could be ascribed to a dual role reported in literature for NR4A2 and IFNγ [pro/anti-inflammatory action (40–43)] and to an involvement also in the detoxification metabolism for NQO1 (59).

Furthermore, secoiridoids and methoxyluteolin especially contributed to the reduction of inflammatory protein expression such as CD14^+^CD16^+^ cell population, and CCL2/CCL4, in agreement with data reported for their molecular expression. On the contrary, 3-hydroxytyrosol, apigenin, and luteolin did not seem to have any significant influence on the expression of these inflammatory signals at protein level. We could only speculate about an early evaluation of EVOO treatment in obese children and/or the role of epigenetic modulations in supporting the discrepancy reported for CCL2/CCL4 protein and molecular data at 6 h. However, since the anti-inflammatory behavior of these three compounds is controversial in literature reports (15, 60, 61), further studies will need to better clarify their ability in reducing the inflammatory response. In this context, it is important to note that several epidemiological studies have shown how following a dietary pattern (i.e., MD) including foods rich in antioxidant and anti-inflammatory compounds, such as polyphenols, carotenoids, and other phytochemicals, is related to reducing the occurrence of risk factors, like hypertension, hypercholesterolemia, hyperglycemia, and obesity, responsible for the Metabolic Syndrome (MetS). Indeed, the secoiridoids and simple phenols typical of EVOO have been recognized to likely exert along with resveratrol, allyl sulfides, phenolic acids, mono- and poly-unsaturated fatty acids, tocopherols and flavonoids, a synergistic positive action on health outcomes in relation to MetS, such as insulin resistance and type 2 diabetes mellitus, endothelial dysfunctions and dyslipidaemic effects, and overall overweight (62). This observation is in line with a current strategy in pharmacology consisting on the identification of molecules acting on the multiple targets usually involved in multi-factorial or complex diseases, such as the non-communicable diseases, to gain access to a clinical benefit (63).

Altogether, our *ex vivo* study gives some advances on the current view of EVOO treatment in the context of obesity because the results refer to a pediatric population, show a good correlation between the chemical composition and the anti-inflammatory activity and confirm our *in vitro* data by using an EVOO extract with the same chemical profile. These important results will prompt us to plan a clinical trial using a secoiridoids-rich EVOO in obese children; the use of the same food matrix could help to reduce the inconsistency usually reported during the translation of data from preclinical to clinical studies. To this aim, it could be also useful to investigate the regulation of adipocytokines by EVOO polyphenols. In fact, adipocytokines are secreted by adipose tissue and act as important modulators of the energy balance and the inflammatory mechanisms (64). Among these, leptin, visfatin, and resistin have a pro-inflammatory role decreasing the insulin sensitivity and inducing the development of chronic complications (65); on the contrary, adiponectin shows anti-inflammatory properties in the context of obesity (66). Even if many human studies investigated the association of adipokines with some clinical parameters [e.g., BMI, waist circumference, and insulin concentration (67–69)], some inconsistencies are reported, likely due to differences in the experimental design, the food matrix, the technical assay used, etc. (10, 13). For this reason, the study of secoiridoids-rich EVOO ability to modulate adipocytokines in the clinical setting of obesity could help to fill the gap with the preclinical data.

However, the limitation of this study is linked to the absence of bioavailability data relative to olive oil extracts. This was mainly due to the multiple variables involved in bioavailability modulation, such as the intestinal and hepatic metabolisms as well as the microbiota metabolism, that require a deep investigation to be conducted separately. To this aim, additional studies are required to have a more integrated view of EVOO as functional food. Specifically, microbiota plays a crucial role in obesity pathogenesis (70). In fact, patients affected by obesity show a significant reduction in gut microbiota diversity and are characterized by a dysbiotic state promoting a low-grade inflammation through different mechanisms mainly linked to intestinal permeability (71). However, thanks to the two-way relationship between polyphenols and microbiota, the consumption of olive oil polyphenols is able to revert the aforementioned pro-inflammatory effects thanks to the polyphenols prebiotic effect and their ability to modulate the production of short chain fatty acids (SCFA) by gut bacteria (72). In fact, SCFA act as critical players of the immune system and could also induce weight loss in obese patients by promoting satiety (73).

Overall, our results could pay the way for the use of secoiridoids-rich EVOO in preventive and/or adjuvant treatments for obesity and severe obesity and, potentially, for the related non-communicable diseases, even if additional studies are needed to verify EVOO anti-inflammatory ability and its correlation to the chemical profile in this pathological context.

## Data availability statement

The gene expression datasets generated and/or analysed during this current study are available from the corresponding author upon reasonable request.

## Ethics statement

The studies involving human participants were reviewed and approved by the Independent Ethical Committee of Azienda Ospedaliera-Universitaria “Consorziale Policlinico” of Bari. Written informed consent to participate in this study was provided by the participants’ legal guardian/next of kin.

## Author contributions

FC, MFF, and SD: conceptualization. SD, LP, AMe, PC, PP, and LA: methodology. SD, PC, NAC, and FC: validation. AMo, MLC, FC, and MFF: resources and funding acquisition. SD and PC: writing—original draft preparation. FC, MLC, PP, LG, and MFF: writing—review and editing. FC, MLC, MFF, and SD: supervision and project administration. All authors have read and agreed to the published version of the manuscript.

## Footnotes

1
www.ingenuity.com
