# Supplementary material for: Extra virgin olive oil extract rich in secoiridoids induces an anti-inflammatory profile in peripheral blood mononuclear cells from obese children
Source: Front Nutr. 2022 Oct 26;9:1017090. doi: 10.3389/fnut.2022.1017090 (PMC9643887; doi:10.3389/fnut.2022.1017090)
Supplement: Supplementary file 1 [file Data_Sheet_1.zip › Suppl. Table S1.docx]

**Supplementary Table 1.** MRM transitions in UHPLC-MS/MS analyses for the quantification of the polyphenols in olive oil samples.

| **Compound** | **[M-H]^−^(*m/z*)^a^** | **MS/MS^b^ fragments** | **MRM transition (*m/z*)** | **EVOO (µg/g)** | **Olive oil (µg/g)** |
| --- | --- | --- | --- | --- | --- |
| 3-hydroxy-tyrosol | 153 | 123, 95 | 153 >> 123 | 136.33 | 2.19 |
| Tyrosol | 137 | 119 | 137 >> 119 | n.d.^e^ | n.d. |
| Oleacinic acid Open Form II | 335 | 199, 155, 111, 59 | 335 >> 199 | n.d. | n.d. |
| Oleuropein aglycone carboxylic acid Open Form I or II | 393 | 257, 169, 111 | 393 >> 257 | tr.^f^ | n.d. |
| Oleuropein isomer 1 | 539 | 113 | 539 >> 113 | 0.70 | n.d. |
| Pinoresinol | 357 | 221 | 357 >> 221 | n.d. | n.d. |
| Oleuropein aglycone enolic-aldehydic Open Form I^c^ | 377 | 275, 149, 139, 121, 111, 101, 95 | 377 >> 275 | 5904.06 | 170.51 |
| Oleuropein isomer 2 | 539 | 113 | 539 >> 113 | 2.36 | n.d. |
| Oleocanthalic acid open form II^c^ | 319 | 199, 181, 155, 139, 121, 111, 85 | 319 >> 199 | 8.73 | n.d. |
| Oleuropein aglycone dialdehydic Open Form I^c^ | 377 | 275, 149, 139, 121, 111, 101, 95 | 377 >> 275 | 7623.85 | n.d. |
| Ligstroside aglycone enolic-aldehydic Open Form I^c^ | 361 | 291, 171, 139, 127, 101, 69 | 361 >> 291 | tr. | n.d. |
| Luteolin | 285 | 217, 199, 175, 133 | 285 >> 133 | 51.65 | 0.69 |
| Hydroxy-methyl decarboxymethyl  ligstroside aglycone isomer 1^c^ | 333 | 181, 111, 99, 94, 69 | 333 >> 181 | tr. | n.d. |
| Ligstroside aglycone enolic-aldehydic Open Form I^c^ | 361 | 291, 171, 139, 127, 101, 69 | 361 >> 291 | 5421.20 | 29.10 |
| Oleuropein aglycone dialdehydic Open Form I^c^ | 377 | 275, 149, 139, 121, 111, 101, 95 | 377 >> 275 | 7754.77 | 229.63 |
| Oleuropein aglycone dialdehydic Open Form I | 377 | 275, 149, 139, 121, 111, 101, 95 | 377 >> 275 | 7719.22 | 224.53 |
| Ligstroside aglycone enolic-aldehydic Open Form I^c^ | 361 | 291, 171, 139, 127, 101, 69 | 361 >> 291 | 4481.56 | 24.71 |
| Apigenin | 269 | 150, 117, 107 | 269 >> 117 | 18.28 | 0.16 |
| Methoxyluteolin^d^ | 299 | 227, 199 | 299 >> 227 | 15.96 | 0.11 |
| Mono enolic-aldehydic oleuropein aglycone Closed Form I^c^ | 377 | 275, 149, 139, 121, 111, 101, 95 | 377 >> 275 | tr. | n.d. |
| Ligstroside aglycone dialdehydic Open Form I^c^ | 361 | 291, 171, 139, 127, 101, 69 | 361 >> 291 | 5105.06 | 27.39 |
| Ligstroside aglycone dialdehydic Open Form I^c^ | 361 | 291, 171, 139, 127, 101, 69 | 361 >> 291 | 5178.28 | 57.06 |
| Mono enolic-aldehydic dihydropyranic ligstroside aglycone Closed Form I^c^ | 361 | 291, 171, 139, 127, 101, 69 | 361 >> 291 | 1773.77 | 21.87 |

^a^ Deprotonated molecule; ^b^ product ions; ^c^ quantified as oleuropein equivalents; ^d^ quantified as luteolin equivalents; ^e^ compound under the LOD; ^f^ compound under the LOQ.
