# Supplementary material for: Extra virgin olive oil extract rich in secoiridoids induces an anti-inflammatory profile in peripheral blood mononuclear cells from obese children
Source: Front Nutr. 2022 Oct 26;9:1017090. doi: 10.3389/fnut.2022.1017090 (PMC9643887; doi:10.3389/fnut.2022.1017090)
Supplement: Supplementary file 1 [file Data_Sheet_1.zip › Suppl. Table S2.docx]

**Supplementary Table 2.** Quantification (mean ± SD) of the identified polyphenols in the PBMCs supernatants at 0, 6, and 24 hours (n = 10).

| **Compound** | **EVOO 0h (µg/mL)** | **Olive oil 0h (µg/mL)** | **EVOO 6h (µg/mL)** | **Olive oil 6h (µg/mL)** | **EVOO 24h (µg/mL)** | **Olive oil 24h (µg/mL)** |
| --- | --- | --- | --- | --- | --- | --- |
| 3-hydroxytyrosol | 2.61 ± 0.15a | 0.043 ± 0.003b | 0.02 ± 0.03b | n.d.^c^ | 0.010 ± 0.019b | n.d. |
| Oleuropein isomer 1 | 0.070 ± 0.003 | tr.^d^ | n.d. | n.d. | n.d. | n.d. |
| Oleuropein aglycone enolic-aldehydic Open Form I^a^ | 30 ± 3a | 1.03 ± 0.10b | n.d. | n.d. | n.d. | n.d. |
| Oleuropein isomer 2 | 0.124 ± 0.010 | tr. | n.d. | n.d. | n.d. | n.d. |
| Oleocanthalic acid open form II^a^ | 0.157 ± 0.004 | n.d. | n.d. | n.d. | n.d. | n.d. |
| Oleuropein aglycone dialdehydic Open Form I^a^ | 39 ± 4 | n.d. | n.d. | n.d. | n.d. | n.d. |
| Luteolin | 1.04 ± 0.03a | 0.015 ± 0.004c | 0.10 ± 0.09b | n.d. | 0.029 ± 0.014bc | n.d. |
| Ligstroside aglycone enolic-aldehydic Open Form I^a^ | 28 ± 3a | 0.269 ± 0.018b | n.d. | n.d. | n.d. | n.d. |
| Oleuropein aglycone dialdehydic Open Form I^a^ | 40 ± 4a | 1.35 ± 0.14b | n.d. | n.d. | n.d. | n.d. |
| Oleuropein aglycone dialdehydic Open Form I | 40 ± 4a | 1.32 ± 0.14b | n.d. | n.d. | n.d. | n.d. |
| Ligstroside aglycone enolic-aldehydic Open Form I^a^ | 23 ± 2a | 0.245 ± 0.015b | n.d. | n.d. | n.d. | n.d. |
| Apigenin | 1.413 ± 0.008a | 0.013 ± 0.005c | 0.040 ± 0.019b | n.d. | 0.029 ± 0.011bc | n.d. |
| Methoxyluteolin^b^ | 1.644 ± 0.007a | 0.012 ± 0.005c | 0.06 ± 0.03b | n.d. | 0.033 ± 0.015bc | n.d. |
| Ligstroside aglycone dialdehydic Open Form I^a^ | 26 ± 3a | 0.260 ± 0.017b | n.d. | n.d. | n.d. | n.d. |
| Ligstroside aglycone dialdehydic Open Form I^a^ | 27 ± 3a | 0.42 ± 0.03b | n.d. | n.d. | n.d. | n.d. |
| Mono enolic-aldehydic dihydropyranic ligstroside aglycone Closed Form I^a^ | 11.5 ± 0.7a | 0.230 ± 0.013b | n.d. | n.d. | n.d. | n.d. |

^a^ Quantified as oleuropein equivalents; ^b^ quantified as luteolin equivalents; ^c^ compound under the LOD; ^d^ compound under the LOQ. Different letters in the same line are significantly different at 5% level (Tukey HSD test).
