# Supplementary material for: Extra virgin olive oil extract rich in secoiridoids induces an anti-inflammatory profile in peripheral blood mononuclear cells from obese children
Source: Front Nutr. 2022 Oct 26;9:1017090. doi: 10.3389/fnut.2022.1017090 (PMC9643887; doi:10.3389/fnut.2022.1017090)
Supplement: Supplementary file 1 [file Data_Sheet_1.zip › Supplementary Figure captions.docx]

**Supplementary Figures captions**

**Supplementary Figure S1**

Gating strategy used to analyze the CD14^+^CD16^+^ cell population from PBMCs in a representative sample. Stained PBMCs were first analyzed to select single cells (dot plot on forward scatter: height vs. area, FSC-H vs FSC-A); live cells were then identified on a dot plot based on the 7-AAD staining vs. side scatter, SSC. To preliminary identify the monocytes population from PBMCs, gated single and live cells were checked for positivity to CD64 and CD45 (each marker vs. SSC). Gated CD64^+^ CD45^+^ cells were analyzed for the expression of CD14 and CD16 by a dot plot.

**Supplementary Figure S2**

MRM analysis of spiked control with phenolic standards: 1) 3-hydroxytyrosol, 2) oleuropein, 3) luteolin, 4) apigenin.
