# Supplementary figures and images for: Extra virgin olive oil extract rich in secoiridoids induces an anti-inflammatory profile in peripheral blood mononuclear cells from obese children
Source: Front Nutr. 2022 Oct 26;9:1017090. doi: 10.3389/fnut.2022.1017090 (PMC9643887; doi:10.3389/fnut.2022.1017090)

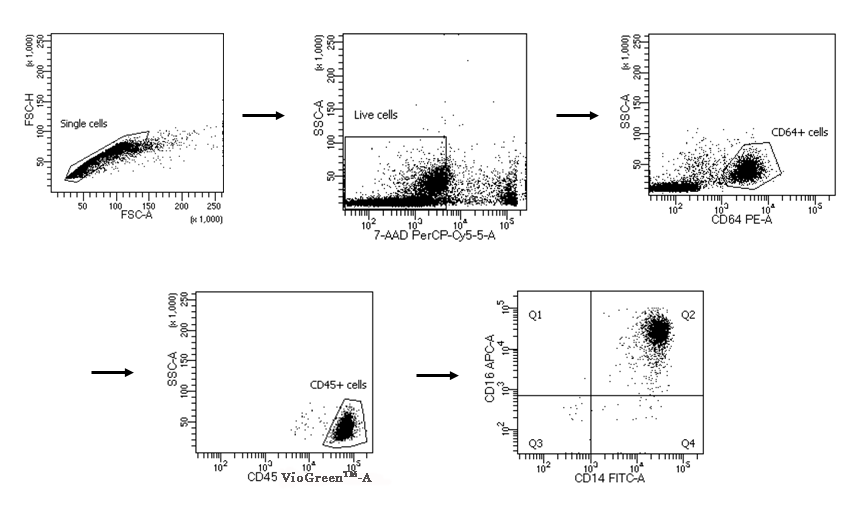

Supplement: Supplementary file 1 [file Data_Sheet_1.zip › Suppl. Figure S1.tif]

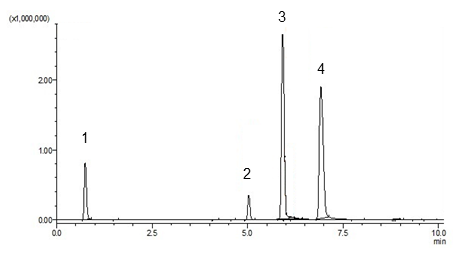

Supplement: Supplementary file 1 [file Data_Sheet_1.zip › Suppl. Figure S2.tif]
